# Structure and Neuroprotector Properties of a Complex Compound of Lithium with Comenic Acid

**DOI:** 10.3390/ijms25010286

**Published:** 2023-12-24

**Authors:** Stanislav Kozin, Alexandr Kravtsov, Lev Ivashchenko, Victor Dotsenko, Stepan Dzhimak, Nicolai Aksenov, Arthur Vashurin, Vasily Ivlev, Mikhail Baryshev, Alexandr Bespalov, Lilia Fedulova, Anna Dorohova, Anastasia Anashkina

**Affiliations:** 1 Physics and Technology Faculty, Kuban State University, 350040 Krasnodar, Russia; stas.fizika@list.ru (S.K.); aakravtsov@mail.ru (A.K.); jimack@mail.ru (S.D.); anna013194@mail.ru (A.D.); 2 Laboratory of Problems of Stable Isotope Spreading in Living Systems, Federal Research Center the Southern Scientific Center of the Russian Academy of Sciences, 344006 Rostov-on-Don, Russia; barishev_mg@mail.ru; 3 Laboratory of Technologies for the Production of Physiologically Active Substances, Kuban State Technological University, 350072 Krasnodar, Russia; 4 Faculty of Chemistry and High Technologies, Kuban State University, 350040 Krasnodar, Russia; chemical000brains@gmail.com (L.I.); victor_dotsenko_@mail.ru (V.D.); bespalov-alex@mail.ru (A.B.); 5 Faculty of Chemistry and Pharmacy, North Caucasus Federal University, 355017 Stavropol, Russia; radioanimation@rambler.ru; 6 Kurnakov Institute of General and Inorganic Chemistry of the Russian Academy of Sciences, 119071 Moscow, Russia; vashurin@isuct.ru; 7 Research and Educational Resource Center “Pharmacy”, RUDN University, 117198 Moscow, Russia; chemistron@mail.ru; 8 Experimental Clinic-Laboratory of Biologically Active Substances of Animal Origin, The V.M. Gorbatov Federal Research Center for Food Systems of Russian Academy of Sciences, 109316 Moscow, Russia; fedulova@vniimp.ru; 9 Engelhardt Institute of Molecular Biology, Russian Academy of Sciences, 119991 Moscow, Russia

**Keywords:** gamma-pyrones, comenic acid, lithium, lithium comenate, lithium complex compounds, neuroprotection, antioxidant, excitotoxicity

## Abstract

The structure, antioxidant and neuroprotective properties of lithium comenate (lithium 5-hydroxy-4-oxo-4H-pyran-2-carboxylate) were studied. Lithium comenate was obtained by reacting comenic acid (H_2_Com) with lithium hydroxide in an aqueous solution. The structure of lithium comenate was confirmed via thermal analysis, mass spectrometry, IR, NMR and UV spectroscopy. The crystal structure was studied in detail via X-ray diffraction. The compound crystallized in a non-centrosymmetric space group of symmetry of the orthorhombic system Pna2_1_ in the form of a hydrate, with three water molecules entering the first coordination sphere of the cation Li^+^ and one molecule forming a second environment through non-valent contacts. The gross formula of the complex compound was established [Li(HCom)(H_2_O)_3_]·H_2_O. It has been established that lithium comenate has a pronounced neuroprotective activity under the excitotoxic effect of glutamate, increasing the survival rate of cultured rat cerebellar neurons more than two-fold. It has also been found that the pre-stress use of lithium comenate at doses of 1 and 2 mg/kg has an antioxidant effect, which is manifested in a decrease in oxidative damage to the brain tissues of mice subjected to immobilization stress. Based on the data available in the literature, we believe that the high neuroprotective and antioxidant efficacy of lithium comenate is a consequence of the mutual potentiation of the pharmacological effects of lithium and comenic acid.

## 1. Introduction

The medical use of lithium ions began in the middle of the 19th century with the treatment of gout with lithium carbonate. Later, there were reports of successful treatment of mania and depression. In the early 1960s, publications appeared indicating the ability of lithium ions to prevent relapses of affective disorders. In the 1970s, their therapeutic activity was shown in depressive episodes, and in the early 1980s, an increase in the effectiveness of antidepressants with lithium ions was shown [1]. For a long time, lithium ions have been used as a therapeutic agent for bipolar disorder [2]. Recent studies have also shown that it has the potential to treat many other neurodegenerative diseases, including Alzheimer’s, Parkinson’s and Huntington’s, due to its neurotrophic, neuroprotective, antioxidant and anti-inflammatory properties [3]. Inorganic and organic ligands are used to deliver lithium ions to the body; however, the use of mineral acid residues has revealed a number of limitations associated with the development of side effects [4]. It has been established that organic molecules have a huge potential for delivering lithium ions to the body [5]. In this regard, it is important to expand the range of possible ligands for lithium ions, which increase its therapeutic potential and reduce its toxic effect.

**Figure 1:**
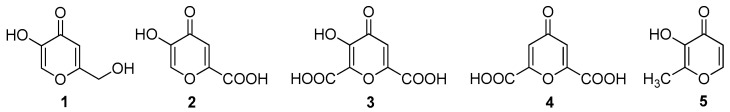
Structural formulas of common γ-pyrones: **1**—kojic acid, **2**—comenic acid, **3**—meconic acid, **4**—chelidonic acid, **5**—maltol.

In this work, we studied the structure, antioxidant and neuroprotective properties of a complex compound of lithium with comenic acid. Comenic acid is a representative of 4H-pyrones (or γ-pyrones). Regarding this class of compounds (Figure 1), there are data in the literature indicating a high therapeutic potential in preclinical studies [6,7,8,9,10,11,12,13,14,15].

It should be noted that comenic acid is an active component of the drug baliz-2, used for cell regeneration in various pathologies [16,17]. Comenic acid reduces the toxic effect of glutamate on the culture of cerebellar neurons [18]. This effect is expressed as a decrease in the level of neuron death and a decrease in the number of calcium ions in the cytosol [18]. In addition to these properties, it has a neurotrophic effect; prevents the disruption of synaptic plasticity of hippocampal neurons under stress; and has antioxidant, moderately anti-amnestic and anxiolytic effects [7,19,20,21].

Thus, the known biological effects of comenic acid suggest that its use as a ligand for lithium ions will produce a synergistic effect. The aim of this work was to study the structure of a lithium complex with comenic acid (lithium comenate) and its antioxidant and neuroprotective properties.

## 2. Results

### 2.1. Chemical Part

**Figure 2:**
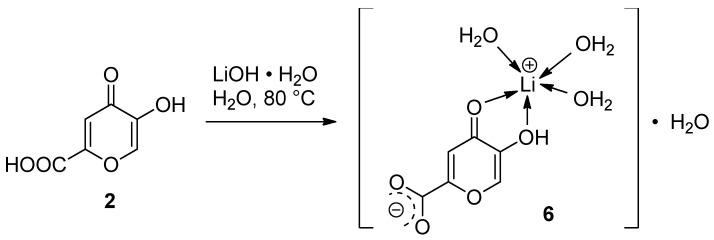
Scheme for preparation of lithium comenate (**6**).

Singly substituted lithium comenate [Li(HCom)(H_2_O)_3_]·H_2_O (**6**) was prepared via the reaction of comenic acid **2** with lithium hydroxide monohydrate when heated in water (Figure 2). The composition of the reaction product was studied in detail using the calculations of simultaneous thermal analysis curves and refined using mass spectrometry data. The molecular structure of the comenate complex was studied using FT-IR spectrometry, ^1^H and ^13^C NMR spectroscopy, UV spectroscopy and thermal analysis and detailed via X-ray diffraction analysis.

#### 2.1.1. Thermal Analysis

Thermal–oxidative degradation and hydrate composition of the lithium coordination compound were studied using synchronous thermogravimetric analysis. Figure S1 (Supplementary File) shows the synchronous thermal analysis curves (TG, DTG and DSC curves) of lithium comenate. Initially, the DSC curve showed a weakly intense endo effect (peak 95.1 °C, −0.10 K), which suggests that at temperatures up to 108.3 °C, sorption moisture was removed from the compound with a mass change of 1.07% according to the TG curve. A weak endo effect flowed into a more intense peak, also of an endothermic nature (peak 160.0 °C, −0.65 K), which suggests that at temperatures up to 173.0 °C, water of crystallization was removed from the compound with a mass loss of 9.34% by weight. The TG curve quantitatively corresponds to one molecule of water, probably outer sphere. Above 322.3 °C, the decomposition of the ligand itself began, which was reflected on the TG curve as a sharp drop characterizing the total weight loss up to 62% and reflected in a series of exo- (peak 354.2 °C, 4.95 K; peak 416.8 °C, 0.24 K) and endo-effects (peak 382.9 °C, −1.61 K; peak 439.5 °C, −1.81 K). Subsequently, the DSC curve showed endothermic peaks (peak 718.9 °C, −0.13 K; peak 796.0 °C, −0.01 K), which are characteristic of the occurrence of oxidative processes in the temperature range from 473 °C to 900 °C; this thermal anomaly was not reproduced when the sample was reheated, which proves the irreversibility of the phase transition, but a mass loss of 5.41% is always observed, reaching 22.18% at 915.7 °C. Gaseous substances were formed as reaction products: CO, CO_2_, H_2_O. The solid residue formed consisted of Li_2_O.

#### 2.1.2. Simultaneous Thermal Analysis/Mass Spectrometry

The lithium comenate sample was additionally analyzed via thermogravimetry and differential scanning calorimetry with vapor phase mass spectral analysis. The results of the synchronous analysis are presented in Table S1 (Supplementary file). The rows of the table correspond to temperature transitions, the columns correspond to the observed signals of the mass spectrometer with the numerical ratio m/z. The cells of the table present the values of the recorded ion current in amperes.

The decomposition of the lithium comenate occurred in several stages. During the first stage (at a temperature of 99 °C), a part of the water was lost; however, in the mass spectrum, the intensity of the peak with a mass number of 18 gradually decreased down to 156 °C. At this temperature, a significant jump in intensity occurred. This degradation step indicated the removal of water from the sample. The next stage of decomposition occurred at temperatures ranging from 339 °C to 354 °C. In this interval, several intense peaks with mass numbers 28, 44 and 112 appeared at once. Mass numbers 28 and 44 corresponded to the release of decomposition products, CO and CO_2_, while mass number 112 probably corresponded to the decarboxylation product of the ligand molecule, 3-hydroxy-4H-pyrone. At the same time, taking into account the data from works [21] regarding the thermal degradation of comenic acid, the formation of an unstable fragment O=C(CO)CH=CH–C=OH^+^ with the same mass number cannot be ruled out. The intensity of the peak with a mass number of 112 was approximately 0.6 × 10^−9^ A, which is more than 10 times less than the value for carbon dioxide (12 × 10^−9^ A). At the same time, the intensity of the peak (m = 112) grew over a short temperature interval and then almost immediately dropped to zero. The intensity of the band with a mass number characteristic of comenic acid (m = 154) slowly increased with increasing temperature and reached a maximum at a temperature of 980 °C. However, even at the maximum of its intensity, this signal was at the detection limit (intensity 0.1 × 10^−9^ A). Comenic acid also broke down into smaller fragments (with a mass less than 112), the peak intensities of which gradually increased with increasing sample temperature (m = 57, 91 and 105). As shown earlier [21], it is difficult to unambiguously determine the degradation products of comenic acid, since a large number of variations that are formed during the degradation of fragments are possible. However, in all cases, the degradation process includes the opening of the pyran ring. Therefore, peaks with mass numbers of 81, 82 or 83 were not observed in the mass spectrum.

#### 2.1.3. IR Spectroscopy

The assignment of characteristic absorption bands in the IR spectrum of 6 and indirect conclusions regarding the structure of the obtained compound 6 (Figure S5, Supplementary File) was carried out by comparing it with the spectrum of compound **2** (Figure S6) and analyzing the literature data on β-hydroxy-γ-pyrones.

On the IR spectrum (Table S2 in the Supplementary File), we observed splitting of the ν_O–H_ band in compound **2** (3339 cm^−1^) for at least 3 bands (3464, 3373, 3323 cm^−1^), which indicates the breaking of intramolecular hydrogen bonds in the initial ligand dimers (absorption bands in the 3000−2400 cm^−1^ region). There were no absorption bands in the spectrum of the complex compound in the 3000−2400 cm^−1^ range, which confirms the above fact. There was a broadening of the band near the ν_C-H_ γ-pyrone structure that overlaps with ν_O–H_ (H_2_O), which was confirmed via thermal analysis data, which showed the presence of a significant number of coordinated water molecules in the complex. On the IR spectrum of the complex (compound **6**), the band of stretching vibrations of the carboxyl group of the starting acid ligand split into ν_as_(COO^−^) 1601 cm^−1^ and ν_sym_(COO^−^) 1354 cm^−1^. The difference was 247 cm^−1^, which is more than 200 cm^−1^ and, therefore, in the complex the ligand is in the ionized form, and the oxygen atoms in the ionized carboxylate anion are not equivalent. The presence of the Li–O bond was confirmed via the appearance of medium-intensity absorption bands in the long-wavelength region at 480, 446 and 405 cm^−1^.

#### 2.1.4. NMR Spectroscopy

The NMR ^1^H spectra of lithium comenate **6** and the initial comenic acid **2** were almost identical, the chemical shifts of proton signals differing by no more than δ 0.05 ppm (Figure S3 in the Supplementary File). On the ^13^C NMR spectra, the difference did not exceed 1.7 ppm (Figure S4 in the Supplementary File).

#### 2.1.5. Electron Spectroscopy in the UV Region

The description of the electronic spectrum of lithium comenate is presented in Table S3 (Supplementary File). Thus, the spectrum of lithium comenate **6** shows no fundamental differences from the spectrum of the original comenic acid **2**.

#### 2.1.6. X-ray Diffraction Analysis

**Figure 3:**
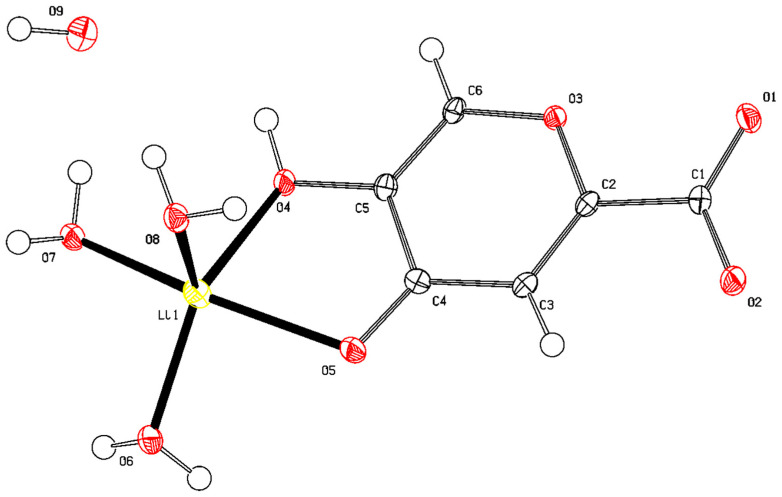
General view of a molecule of a complex compound of lithium with 5-hydroxy-4-oxo-4H-pyran-2-carboxylic acid in a crystal. Lithium atom is colored yellow, carbons are black and white, hydrogens are white, and oxygens are red.

Orthorhombic crystals suitable for single-crystal X-ray diffraction studies were obtained via recrystallization of lithium comenate from an aqueous solution. Complex compound 6 crystallized with four water molecules, and three water molecules completed the first coordination environment of the Li+ cation when it was coordinated through a bidentate fragment of β-hydroxy-γ-pyrone-comenic acid, and another molecule was also bound via short intermolecular contacts and began to form the second coordination sphere of the Li^+^ cation. The ligand molecule was ionized by a carboxyl group. Molecular structure [Li(HCom)(H_2_O)_3_]·H_2_O is shown in Figure 3 and Figure S2 (Supplementary File).

**Figure 4:**
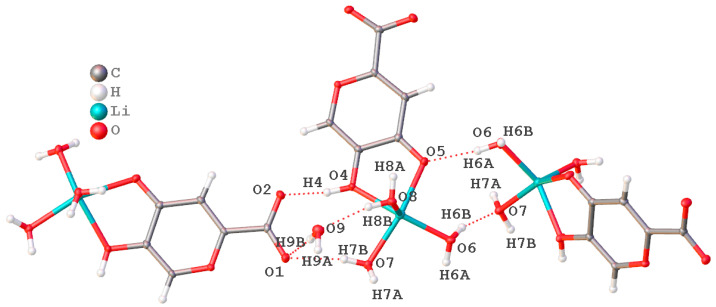
Non-valent intermolecular contacts.

The packing of molecules in the crystal lattice was stabilized by a branched system of hydrogen bonds with the participation of solvate water molecules and oxygen atoms of various functional groups of ionized ligand molecules (Figure 4). The main crystallographic characteristics and parameters of X-ray diffraction experiments for the compound are given in Table S4 (Supplementary File). The most important bond lengths and bond angles within the coordination polyhedron are presented in Tables S7–S9 (Supplementary File).

The Li^+^ ion was in a five-coordinated oxygen environment formed by intrasphere-coordinated water molecules, as well as atoms of the hydroxyl (C3) and carbonyl groups (C4) of the ligand. The coordination sphere of Li^+^ was found to be close to square pyramidal with geometry index τ = 0.159. The compound crystallizes in the non-centrosymmetric space group of symmetry of the orthorhombic system Pna2_1_ as a hydrate.

Atomic coordinates and other structure parameters (Tables S5, S6, S10 and S11 (Supplementary File)) were deposited at the Cambridge Crystallographic Data Center (CCDC № 2288394); www.ccdc.cam.ac.uk/structures, accessed on: 1 September 2023.

### 2.2. Biological Part

#### 2.2.1. Neuroprotective Activity during Excitotoxic Exposure

**Figure 5:**
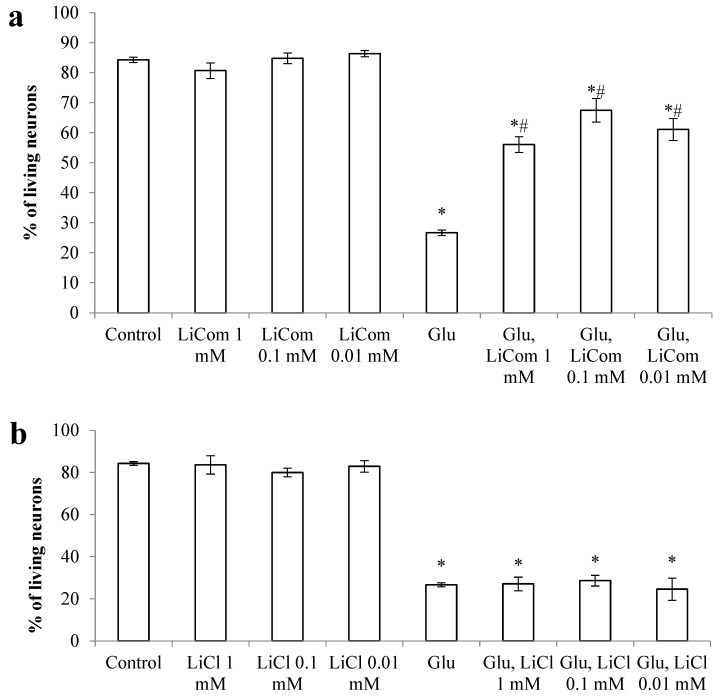
Effect of lithium comenate (LiCom, **a**) and lithium chloride (LiCl, **b**) on neuronal survival after exposure to glutamate (Glu). * *p* < 0.05 in relation to control, # *p* < 0.05 in relation to Glu.

The results of studies of the neuroprotective properties of lithium comenate showed that the number of surviving neurons, when exposed to both lithium chloride and lithium comenate, in the absence of Glu, practically did not differ from the control (Figure 5). After exposure to Glu, the survival of neurons significantly decreased (up to 27%). The survival rate of neurons remained practically at the same level after the addition of lithium chloride after exposure to Glu in all studied concentrations. At the same time, the use of lithium comenate contributed to a significant reduction in neuronal death after excitotoxic exposure. Thus, in all groups of cultures with the addition of lithium comenate against the background of the use of Glu, the survival of neurons was, on average, 2 times higher than in the group with Glu. The maximum efficiency was noted at a concentration of 0.1 mM. Thus, the use of lithium comenate after exposure to Glu contributes to a significant reduction in the death of cerebellar neurons in culture.

Lithium chloride in this model did not affect the resistance of neurons to Glu.

**Figure 6:**
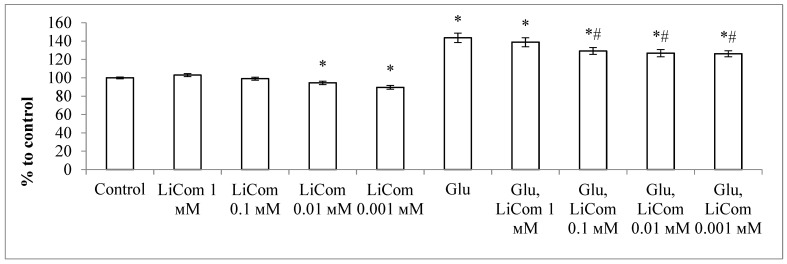
Effect of lithium comenate (LiCom) on the intraneuronal Ca^2+^ level after excitotoxic exposure to glutamate (Glu). * *p* < 0.05 in relation to control, # *p* < 0.05 in relation to Glu.

A study using Fluo-4 showed that Glu exposure led to an increase in intraneuronal Ca^2+^ by 44% relative to control (Figure 6). In cultures with the addition of lithium comenate at concentrations of 0.1–0.001 mM, the Ca^2+^ level increased significantly to a lesser extent, by 26–29% compared to the control. Thus, the use of lithium comenate at concentrations of 0.1–0.001 mM after exposure to Glu largely prevented an increase in the level of Ca^2+^ in cultured neurons.

#### 2.2.2. Influence of Lithium Comenate on the Prooxidative and Antioxidant Status of the Brain under Stress

**Figure 7:**
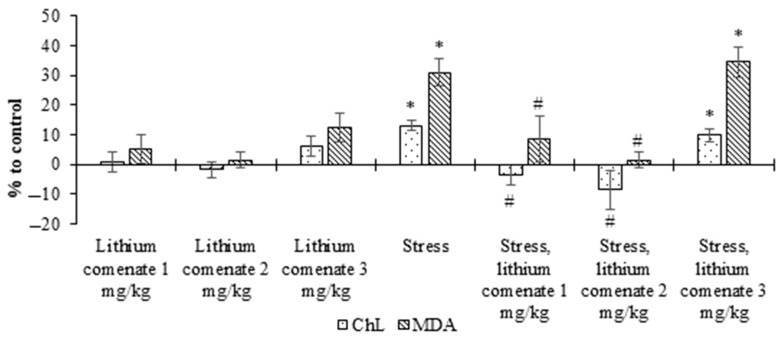
Effect of lithium comenate on chemiluminescence intensity (ChL) and malondialdehyde (MDA) content in the brains of stressed mice during pre-stress application. * *p* < 0.05 in relation to control, # *p* < 0.05 in relation to stress.

The analysis of the obtained data (Figure 7) showed that the immobilization stress effect promoted the activation of oxidative processes in the brains of mice, which manifested in a significant increase in chemiluminescence intensity and malondialdehyde (MDA) level.

After the administration of lithium comenate at doses of 1 and 2 mg/kg, a statistically significant decrease in ChL intensity and MDA content was noted in the brains of stressed mice. At the same time, the most pronounced decrease in the intensity of ChL and MDA was observed in the group of animals treated with lithium comenate at a dose of 2 mg/kg (*p* ≤ 0.001). Lithium comenate at a concentration of 3 mg/kg did not have any effect on oxidative processes in the brain during stress. Thus, in the group of stressed mice treated with lithium comenate at a dose of 3 mg/kg, the ChL intensity and the level of MDA in the brain remained practically at the level of stressed animals. Also, with the introduction of lithium comenate at a dose of 3 mg/kg to intact animals, a statistically insignificant activation of lipid peroxidation (LPO) was observed—an increase in the level of MDA.

Thus, it has been established that the stress effect on the body of experimental animals is accompanied by an increase in oxidative processes in the brain, which manifest in an increase in the intensity of the ChL reaction and an increase in the content in the brain of one of the secondary LPO products, MDA. The use of lithium comenate at doses of 1 mg/kg and 2 mg/kg has an antioxidant effect, which manifests in preventing the hyperproduction of free radicals and increasing the content of lipid peroxidation products in the brains of stressed animals.

## 3. Discussion

The excitotoxic effect is largely mediated through glutamate receptors known as NMDA (N-methyl-D-aspartate) receptors, as well as through AMPA (α-amino-3-hydroxy-5-methyl-4-isoxazolepropionic acid) receptors. Upon binding to these receptors, there is an influx of calcium (Ca^2+^) into the neuron. This process is one of the key mechanisms of damage to and death of neurons. It is known that excitotoxicity and oxidative stress enhance each other’s pathological effects, being links in the same pathological process. An excess of Ca^2+^ enhances the activity of Ca^2+^-dependent enzymes, such as neuronal NO synthase [22]. The high activity of this enzyme leads to hyperproduction of reactive nitrogen species and intensification of lipid peroxidation processes. An increase in cytosolic calcium ions activates phospholipases and kinases involved in starting the apoptosis process [23].

In our experiments, it was established that lithium comenate prevents an increase in the level of Ca^2+^ in neurons after excitotoxic exposure to glutamate and exhibits a pronounced neuroprotective activity, which manifests in an increase in the survival of neurons by 30–40%. The protective effect of lithium comenate on the culture of cerebellar neurons in our study can be explained by the biological effects of lithium and comenic acid residue (comenate). In one of our past works [7], we showed that comenic acid has the effect of a direct antioxidant, participating in the inhibition of free radical oxidation processes in model systems. The presence of a hydroxyl residue in the third position and the pyran ring makes it possible for the comenic acid residue to neutralize free radicals and suppress the development of oxidative stress, reducing the toxic effect of glutamate. It should be noted that the protective effect of comenic acid during excitotoxic exposure, which we studied earlier [7], was significantly lower than that of lithium comenate and is about 20% at a concentration of 1 mM and about 10% at a concentration of 0.1–0.01 mM. At the same time, lithium chloride in our experiments did not have a neuroprotective effect. Other authors have found a protective effect of lithium chloride against glutamate toxicity in cultured cerebellar neurons. However, the neuroprotective effect of this drug manifested only after preliminary incubation of cultures with 1–5 mM LiCl for 3–7 days [24]. These data suggest that lithium, by triggering a number of signaling processes, can potentiate the antioxidant defense of cells, which is consistent with the results of our in vivo experiments, as well as the data of other authors cited above [25]. Activation of the GSK-3β enzyme leads to apoptotic death of neurons caused by various neural injuries, including the excitotoxic effects of glutamate [26]. It is likely that the neuroprotective effect of lithium during chronic use is due mainly to its ability to directly or indirectly inhibit the kinase activity of GSK-3β [27]. The transcription factor β-catenin is a GSK-3β substrate and part of the Wnt pathway. Its cytoplasmic levels are negatively regulated by constitutively active GSK-3β. An increase in cytoplasmic accumulation of β-catenin mediated by inhibition of GSK-3β facilitates its translocation into the nucleus and triggers the expression of genes for a number of protective proteins BDNF, VEGF, Bcl2 and HSP-70 [28]. There is evidence that lithium, by inactivating the Src kinase, inhibits the phosphorylation of NR2B, a subunit of the NMDA receptor, which leads to its inactivation [29,30] and inhibits NMDA receptor expression in rats [31]. In addition, it has been shown that Li^+^ inhibits the sodium–calcium exchanger, increasing intracellular Ca^2+^ levels, and enhances calcium-dependent desensitization of the NMDA receptor [32,33]. In the study [24], it was found that short-term (1 h) treatment of cultures with lithium did not affect glutamate-induced Ca^2+^ entry into neurons, while long-term treatment (7 days) significantly reduced glutamate-induced Ca^2+^ entry into neurons.

In our study, cerebellar neurons were exposed to a short (two-hour) action of lithium comenate. This was expressed by a decrease in the cytosolic level of Ca^2+^ with the addition of lithium comenate at concentrations of 0.1–0.001 mM after exposure to glutamate, while at a concentration of 1 mM, the level of Ca^2+^ did not change significantly. Also, at concentrations of 0.01 and 0.001 mM in cultures not exposed to glutamate, the Ca^2+^ level decreased, while at 1 and 0.1 mM it did not change.

This exposure time is insufficient for lithium ions to activate protective signaling pathways, such as those in which GSK-3β is involved. The protective effect of lithium observed in our study is probably associated with a direct effect on the Na^+^/Ca^2+^ exchanger. 

## 4. Materials and Methods

IR spectra were recorded on a Bruker Vertex 70 IR Fourier spectrometer (Bruker, Karlsruhe, Germany) with an ATR attachment on a diamond crystal, spectral resolution ± 4 cm^−1^. NMR spectra were recorded on a Bruker Ascend 700 (700 MHz on ^1^H nuclei, 176 MHz-^13^C) device (Karlsruhe, Germany) in D_2_O solution at 298 K. Residual solvent signals were used as a standard (HDO, δ 4.79 ppm). Electronic spectra were recorded on a two-beam spectrophotometer U-2900 (Hitachi, Tokyo, Japan) in quartz cuvettes (*l* = 10 mm) in the spectral range 190–400 nm. Empirical spectra were smoothed using fast Fourier transforms (FFTs) using the software package OriginLab 2019 (OriginLab Corporation, Northampton, MA, USA).

Thermogravimetric analysis was carried out using a thermomicrobalance (TG 209 F1 Iris from Netzsch) (Selb, Germany) on a differential scanning calorimeter (DSC 204 F1 Phoenix with μ-sensor from Netzsch) (Selb, Germany). The experiment was carried out in an oxidizing atmosphere (air) in alundum crucibles under conditions of programmed isothermal heating with a standard *α*-Al_2_O_3_ at heating rate 10 °C/min and temperature range 30–900 °C. Synchronous thermal analysis of the samples was carried out using thermogravimetry and differential scanning calorimetry with mass spectral analysis of the vapor phase on a thermal analyzer (DSC/DTA/TG) from Netzsch, with a skimmer mass spectrometric system for analyzing the vapor phase.

The purity of the compounds obtained and the course of the reaction were monitored via TLC on Sorbfil PTSKh-AF-A plates (manufactured by IMID Ltd., Krasnodar, Russia), with an eluent of 1:1 acetone–hexane, an iodine vapor developer and a UV detector. For the synthesis, we used as starting compounds LiOH·H_2_O (>99.99%) and comenic acid. All experiments were performed using bidistilled water.

Comenic acid (5-hydroxy-4-oxo-4H-pyran-2-carboxylic acid) was obtained with a yield of 80% via oxidation of glucose using strain 003 of bacteria *Gluconobacter oxydans* according to known methods [34,35] followed by purification via column chromatography and yielded the following IR spectrum measurements (ν, cm^−1^): 3339 (O8-H H_2_L), 3089 (C-H), 2997-2467 (O-H H_2_O), 1726 (C4=O7), 1628 (C=C), 1599 (C1=O), 1420 (C5-O), 1219 (C1-O), 1204 (C5-O-H). NMR spectrum ^1^H [D_2_O, 298 K], (δ, ppm): 7.03 s (1H, C3-H), 8.02 s (1H, C6-H). NMR spectrum ^13^C [D_2_O, 298 K], (δ, ppm): 176.9 (C4), 164.2 (COOH), 156.5 (C2), 146.4 (C5), 142.3 (C6), 115.3 (C3).

The synthesis of monosubstituted 5-hydroxy-4-oxo-4H-pyran-2-carboxylate (lithium comenate) was performed as follows: a solution of 1.00 g (6.4 mmol) of comenic acid in 25 mL of water at a temperature 80 ± 2 °C treated with a solution of 0.27 g (6.4 mmol) LiOH·H_2_O in 5 mL of water. As a result, the reaction mass acquired a pH value of 4.6 and turned bright yellow. Lithium comenate was isolated by evaporating the solution to ~3/4 of the initial volume under reduced pressure. At the same time, lithium comenate began to crystallize from a saturated solution on the next day. The resulting product was further purified via recrystallization from bidistilled water. The yield of lithium comenate was 1.20 g (80%). IR spectrum (ν, cm^−1^): 3612 (O-H H_2_L), 3464, 3374, 3125, 3080 (C-H), 1600 ν*_as_*(COO^−^), 1464, 1408, 1377, 1355 ν*_s_*(COO^−^), 1261, 1203, 1153, 1103, 939, 890, 821, 804, 768, 746, 663, 614, 560, 519, 480, 446, 405.

NMR spectrum ^1^H [D_2_O, 298 K], (δ, ppm): 7.02 s (1H, C3-H), 8.07 s (1H, C6-H). NMR spectrum ^13^C [D_2_O, 298 K], (δ, ppm): 177.0, 165.1, 158.2, 146.0, 141.8, 114.3.

X-ray diffraction analysis of a lithium comenate hydrate crystal (C_6_H_11_LiO_9_) performed on an automatic four-circle diffractometer SuperNova, Dual, Cu at zero, Atlas S2 (Agilent Technologies XRD Products, Yarnton, Oxfordshire, UK) at 293 K. The structure was identified using the Olex2 (version v1.1.5, OlexSys Ltd., Durham, UK) and SHELXT software (George M. Sheldrick, Institute of Inorganic Chemistry, Göttingen, Germany, https://www.shelxle.org/shelx/eingabe.php, accessed on 31 November 2023.) packages. The remaining non-hydrogen atoms were localized via a direct method via successive calculations of difference Fourier maps. The positions of the atoms were refined via full-matrix least squares using F^2^*_hkl_* in anisotropic approximation for all non-hydrogen atoms using SHELXL [36]. The contributions of hydrogen atoms were taken into account in the calculations but were not refined. In all cases, the locations of the largest peaks, as well as the values of the residual electron density in the final difference Fourier maps, were chemically insignificant.

The main characteristics of the experiment and the parameters of the unit cell: orthorhombic syngony, space group Pna2_1_, M = 234.09 g/mol, *a* = 6.99950(6) Å, *b* = 21.05846(19) Å, *c* = 6.77637(7) Å, *α* = 90°, *β* = 90°, *γ* = 90°, V = 998.828(16) Å^3^, Z = 4, μ(Cu Kα) = 1.317 mm^−1^, D*_solved_* = 1.557 g/cm^3^, F(000) = 488.0, shooting angle area θ = 8.398−152.766°; reflection index intervals: −8 ≤ h ≤ 8, −26 ≤ k ≤ 26, −8 ≤ l ≤ 7, number of measured reflections 9325, number of independent reflections 1872 (R_int_ = 0.0240, R_sigma_ = 0.0145). R-factors [*I* > 2*σ*(*I*)]: R_1_ = 0.0228 (wR_2_ = 0.0612), R-factors for all reflections: R_1_ = 0.0229 (wR_2_ = 0.0613); GOOF at F^2^ 1.132, Δρ_max_/Δρ_min_ = 0.27/−0.20 e Å^−3^. The XRD results of the compound were deposited at the Cambridge Crystallographic Data Center (CCDC № 2288394).

### 4.1. Study of Neuroprotective Activity under Excitotoxic Exposure

For experiments with cultures of neurons, we used 7–8-day-old cultures of cerebellar neurons obtained via the method of enzymatic–mechanical dissociation from the brains of 7–9-day-old Wistar rats as described in article [18] with some modifications [37]. After sacrificing the pups with ethyl ether, they were treated with 70% ethyl alcohol. After that, the brains were removed and placed on the horizontal surface of the Maximov chamber. Next, the brains were washed with Ca^2+^- and Mg^2+^-free PBS, and the cerebellum was removed. Then the cerebellum was transferred into the well of a Maximov chamber containing 2–3 mL of PBS and excised with a scalpel. The tissues were exposed for 20 min to trypsin (0.05%) and EDTA (0.02%) dissolved in PBS solution at 36.5 °C. After that, the tissue was washed three times with PBS r and once in the culture medium and then subjected to mechanical dissociation in culture medium of the following composition: 90% Minimum Essential Medium, 10% fetal calf serum, 2 mM glutamine, 10 mM HEPES buffer, and 5 mM KCl. The cell suspension was centrifuged at 1500 rpm for 1 min. After that, the supernatant was removed, and the precipitate was resuspended in the nutrition medium with 25 mM KCl necessary for survival of the cerebellum granular neurons. The cultures were grown in 96-well plates coated with poly-L-lysine. To each well, 0.1 mL cell suspension was added for a final density of 3–5 × 10^3^ cell/mm^2^. The cells were cultured in a CO_2_ incubator at 36.5 °C and 98% relative humidity.

The experiments used 7-day cultures of cerebellar neurons. Exposure to glutamate (100 μM) was carried out in a balanced salt solution (BSS) of the following composition (mM): NaCl-154, KCl-25, Na_2_HPO_4_-0.35, CaCl_2_-2.3, NaHCO_3_-3.6, glucose-5.6 and HEPES-5 (pH 7.5). The duration of exposure was 10 min, control cultures were placed for 10 min in BSS without glutamate. After that, the cultures were returned to the original culture medium and placed in a CO_2_ incubator for 4.5 h. Lithium comenate and lithium chloride were introduced into the cultures immediately after their return to the culture medium at final concentrations from 1 to 0.001 mM.

The level of neuron death was determined via morphological analysis of cultures on an inverted microscope Invertoscopes ID 03 (Carl Zeiss, Oberkochen, Germany), which took into account the number of living and dead neurons in 3–5 fields of view. The cultures were preliminarily fixed with a special solution (alcohol 70%, formalin 20%, glacial acetic acid 10%) and stained with trypan blue. The results were presented as the percentage of intact neurons. Statistical analysis of the results was performed using the Mann–Whitney U test in Statistica 10. Data are presented as M ± m. Differences were considered significant at *p* < 0.05.

To study the effect of lithium comenate on the intracellular Ca^2+^ level in glutamate toxicity, we used the Fluo-4 AM fluorescent probe, which is cleaved inside the cell to Fluo-4, which interacts with free calcium and produces fluorescence, the registration of which makes it possible to study the Ca^2+^ level in neurons. Fluo-4 AM was added to the culture medium for 30 min at a concentration of 5 μM. Glutamate exposure was carried out in saline (mM): NaCl-154; KCl-25; Na_2_HPO_4_·12H_2_O-0.35; CaCl_2_-2.3; NaHCO_3_-3.6; glucose-5.6; and HEPES-5 (pH 7.5). The duration of exposure to glutamate was 10 min; the concentration was 100 μM. After 10 min, the cultures were washed with saline and transferred either to saline with test substances or to saline without. Control cultures were placed for 10 min in saline without glutamate. Fluorescence intensity was measured 60 min after exposure to glutamate on a Filter Max F5 multifunctional microplate reader at an excitation wavelength of 485 µm and an emission wavelength of 535 µm. Cultures were washed three times with saline before measurement. The measurement results are presented in %; fluorescence intensity of control cultures was taken as 100%.

### 4.2. Study of the Oxidative Processes in Brain Tissues under Stress

The experiments were carried out on 72 outbred male mice weighing 23–25 g. The following groups of outbred male mice were formed:

1—control;

2—lithium comenate 1 mg/kg;

3—lithium comenate 2 mg/kg;

4—lithium comenet 3 mg/kg;

5—stress;

6—stress, lithium comenate 1 mg/kg;

7—stress, lithium comenate 2 mg/kg;

8—stress, lithium comenate 3 mg/kg.

Lithium comenate was administered to animals orally at doses of 1, 2 and 3 mg/kg of body weight for 3 days, once a day before stress exposure. During the experiment, the animals were kept in standard conditions, with free access to water and food.

The conditions of the animals were standardized: temperature: 20 ± 3 °C, humidity: 48 ± 2%, lighting mode: day/night (from 6.00 a.m. until 6.00 p.m./from 6.00 p.m. until 6.00 a.m.). Birch chips were used as bedding. During the whole experiment, the animals consumed standard concentrated mixed grain. The experiments were carried out in accordance with the requirements of the “Guide for the Care and Use of Laboratory Animals” [38], European Community Directives 2010/63/EU and “Guide for Working with Laboratory Animals, Including the Ethical Principles of Animal Testing (3R principle) of the V.M. Gorbatov Federal Research Center for Food Systems of the Russian Academy of Sciences”. The study was approved by the bioethical commission of the V.M. Gorbatov Federal Research Center for Food Systems of the Russian Academy of Sciences (Protocol No. 7/2023 on 7 March 2023).

The antioxidant activity of lithium comenate was studied in the model of acute immobilization stress. Immobilization stress in mice was induced by hanging them by the neck crease for five hours.

Five hours after the stress, the animals were anesthetized, after which the mice were decapitated. The brain was removed and washed in cold phosphate buffer pH = 7.45 and placed in liquid nitrogen.

The intensity of oxidative processes in the brain tissues of rats was determined using the chemiluminescent method and via the content of malondialdehyde (MDA) [37]. Chemiluminescent analysis was carried out on a Lum 100 instrument (DISoft LLC, Moscow, Russia). The results of the experiments were evaluated based on the intensity of chemiluminescence in c.u. The content of malondialdehyde (MDA) was determined spectrophotometrically from the amount of colored product obtained using interaction with thiobarbituric acid at a wavelength of 532 nm.

Statistical analysis of the results was performed using Kruskal-Wallis rank analysis of variance, Mann–Whitney U test in Statistica 10. Data are presented as M ± SE. Differences were considered significant at *p* < 0.05.

## 5. Conclusions

In this work, a chemical synthesis was performed and the structure of a new substance, lithium comenate (lithium 5-hydroxy-4-oxo-4H-pyran-2-carboxylate), was established. The gross formula of the complex compound was established [Li(HCom)(H_2_O)_3_]·H_2_O. Its antioxidant and neuroprotective properties were studied. The compound crystallizes in a non-centrosymmetric space group of symmetry of the orthorhombic system Pna2_1_ in the form of a hydrate, with three water molecules entering the first coordination sphere of the cation Li^+^, and one molecule forming a second environment through non-valent contacts. Lithium comenate protects cerebellar neurons under the action of toxic doses of glutamate, increases their survival and reduces the level of intracellular calcium. It was also found that the pre-stress use of lithium comenate at doses of 1 and 2 mg/kg has an antioxidant effect, which manifests in a decrease in oxidative damage to the brain tissues of mice subjected to immobilization stress.

Thus, we believe that the neuroprotective efficacy of lithium comenate in experiments with excitotoxic effects is, as in in vivo experiments, the result of mutual potentiation of the pharmacological effects of lithium and comenic acid. The available data indicate the prospects for further studies of lithium comenate as an antioxidant, stress- and neuroprotective pharmacological agent.

## Data Availability

Data are contained within the article.

## Supplementary Materials

The following supporting information can be downloaded at: https://www.mdpi.com/article/10.3390/ijms25010286/s1.
