# Supplementary material for: Structure and Neuroprotector Properties of a Complex Compound of Lithium with Comenic Acid
Source: Int J Mol Sci. 2023 Dec 24;25(1):286. doi: 10.3390/ijms25010286 (PMC10778946; doi:10.3390/ijms25010286)

CONTENTS

Table S1. Results of simultaneous thermal analysis/mass spectrometry of a sample of lithium comenate hydrate. 2

Table S2. Characteristic frequencies (cm^{-1}) and their assignments in the FTIR spectra of comenic acid (1) and lithium comenate (2) 2

Table S3. Parameters of absorption bands in the electronic spectrum lithium comenate....2

Table S4. Crystallographic data, experimental parameters and refinement of the lithium comenate tetrahydrate structure (according to X-Ray results) 3

Table S5. Fractional Atomic Coordinates ($\times10^4$) and Equivalent Isotropic Displacement Parameters (Å2$\times10^3$) for $[Li(HCom)(H_2O)_3]\cdot H_2O$ 4

Table S6. Anisotropic Displacement Parameters ($Å^2\times10^3$) for $[Li(HCom)(H_2O)_3]\cdot H_2O$. 4

Table S7. Bond Lengths for lithium comenate tetrahydrate $[Li(HCom)(H_2O)_3]\cdot H_2O$ 5

Table S8. Bond angles for lithium comenate tetrahydrate $[Li(HCom)(H_2O)_3]\cdot H_2O$....5

Table S9. Torsion Angles for lithium comenate tetrahydrate $[Li(HCom)(H_2O)_3]\cdot H_2O$....5

Table S10. Hydrogen Atom Coordinates (Å$\times10^4$) and Isotropic Displacement Parameters ($Å^2\times10^3$) for lithium comenate tetrahydrate $[Li(HCom)(H_2O)_3]\cdot H_2O$ 6

Table S11. Hydrogen Bonds for lithium comenate tetrahydrate $[Li(HCom)(H_2O)_3]\cdot H_2O$ 6

Figure S1. TG-, DTG-, DSC-curves of a complex compound $[Li(HCom)(H_2O)_3]\cdot H_2O$ 7

Figure S2. Non-valent intermolecular contacts in a lithium comenate crystal....8

Figure S3. ^{1}H NMR (400 MHz, D_2O) spectrum of $[Li(HCom)(H_2O)_3]\cdot H_2O$ 8

Figure S4. ^{13}C NMR (101 MHz, D_2O) spectrum of $[Li(HCom)(H_2O)_3]\cdot H_2O$ 8

Figure S5. FTIR spectrum of comenic acid (ATR mode) 9

Figure S6. FTIR spectrum of complex compound $[Li(HCom)(H_2O)_3]\cdot H_2O$ (ATR mode)....10

Table S1. Results of simultaneous thermal analysis/mass spectrometry of a sample of lithium comenate hydrate.

m/z / T,°C	2 (H_2)	18 (H_2O)	28 (CO)	44 (CO_2)	57	91	105	112	154
99.1	–	–	–	–	–	–	–	–	–
156.7	–	$<40\cdot10^{-9}$	–	–	–	–	–	–	–
339-354	–	–	$12\cdot10^{-9}$	$12\cdot10^{-9}$	–	–	–	$0.6\cdot10^{-9}$	–
413.1	–	–	–	–	–	–	–	–	–
<600	$5\cdot10^{-9}$	–	$20\cdot10^{-9}$	–	$0.5\cdot10^{-9}$	$0.4\cdot10^{-9}$	$0.4\cdot10^{-9}$	–	$0.1\cdot10^{-9}$

Table S2. Characteristic frequencies (cm^{-1}) and their assignments in the FTIR spectra of comenic acid (1) and lithium comenate (2)

Compound	ν O-H	ν C-H	ν O-H (H_2O)	ν C=O (>C^4=O)	ν C=O (>COOH)	ν_{as} COO^-	ν_{sy} COO^-	$\Delta\nu = \nu_{as} COO^- - \nu_{sy} COO^-$	ν C-O-C (γ-pyron)	ν C-O	ν C-OH	δ C-OH	ν M-O
**1**	3338	3086	3000-2450	1726	1628	–	–	–	1219	1203	1144	1099	–
**2**	3612-3323	3080	–	–	–	1601	1354	247	1261	1203	1153	1103	405

Table S3. Parameters of absorption bands in the electronic spectrum lithium comenate

**λ max, nm**	**Conjugate system**	**Transition**	**A, rel. units**	**ε, l/mol·cm**	**log ε**
**200.2**	–COOH	n→π*	0.1980	19498	4.29
**221.6**	=C–O–C=	n→π*	0.2348	23442	4.37
**243.6**	>C=O	n→π*	0.0689	7079	3.85
**282.0**	–OH	π→π*	0.0617	6166	3.79

Table S4. Crystallographic data, experimental parameters and refinement of the lithium comenate tetrahydrate structure (according to X-Ray results)

Empirical formula	$C_6H_{11}LiO_9$
Formula weight	234.09
Temperature/K	100(2)
Crystal system	orthorhombic
Space group	$Pna2_1$
a/Å	6.99950(6)
b/Å	21.05846(19)
c/Å	6.77637(7)
α/°	90
β/°	90
γ/°	90
Volume/Å^3	998.828(16)
Z	4
ρ_{calc}g/cm^3	1.557
μ/mm^{-1}	1.317
F(000)	488.0
Crystal size/mm^3	0.739 × 0.464 × 0.19
Radiation	Cu Kα (λ = 1.54184)
2Θ range for data collection/°	8.398 to 152.766
Index ranges	$-8 \le h \le 8$, $-26 \le k \le 26$, $-8 \le l \le 7$
Reflections collected	9325
Independent reflections	1872 [R_{int} = 0.0240, R_{sigma} = 0.0145]
Data/restraints/parameters	1872/8/152
Goodness-of-fit on F^2	1.131
Final R indexes [I>=2σ (I)]	R_1 = 0.0228, wR_2 = 0.0612
Final R indexes [all data]	R_1 = 0.0229, wR_2 = 0.0613
Largest diff. peak/hole / e Å^{-3}	0.27/-0.20
Flack parameter	0.06(7)

Table S5. Fractional Atomic Coordinates (×10^4) and Equivalent Isotropic Displacement Parameters (Å2×10^3) for [Li(HCom)(H_2O)$_3$]·H_2O. U_{eq} is defined as 1/3 of of the trace of the orthogonalised U_{IJ} tensor.

**Atom**	***x***	***y***	***z***	**U(eq)**
O1	-634.4(17)	1352.7(5)	7897(2)	14.4(3)
O2	-1642.5(18)	2180.6(5)	9702(2)	15.5(3)
O3	338.4(15)	2116.0(5)	4975.6(19)	10.1(3)
O4	1608.0(17)	3516.8(5)	2136(2)	13.2(3)
O5	243.0(17)	4044.4(5)	5382(2)	14.3(3)
O6	841.4(18)	5282.6(5)	3676(2)	13.7(3)
O7	2849.8(16)	4654.3(5)	284(2)	12.8(3)
O8	4726.9(17)	4339.6(5)	4379(2)	13.2(3)
O9	6918.8(18)	3885.2(6)	1245(2)	18.3(3)
C1	-927(2)	1932.1(7)	8200(3)	11.1(3)
C2	-347(2)	2397.8(8)	6611(3)	10.0(3)
C3	-459(2)	3036.1(7)	6826(3)	11.1(3)
C4	211(2)	3450.1(7)	5289(3)	10.8(3)
C5	943(2)	3130.0(7)	3561(3)	10.7(3)
C6	972(2)	2485.2(8)	3464(2)	10.5(3)
Li1	2094(4)	4447.8(13)	3168(5)	14.6(6)

Table S6. Anisotropic Displacement Parameters (Å^2×10^3) for [Li(HCom)(H_2O)$_3$]·H_2O. The Anisotropic displacement factor exponent takes the form: $-2\pi^2[h^2a^{*2}U_{11}+2hka^*b^*U_{12}+\ldots]$.

**Atom**	U_{11}	U_{22}	U_{33}	U_{23}	U_{13}	U_{12}
O1	21.1(6)	10.3(5)	11.7(7)	1.8(5)	2.0(5)	-0.4(4)
O2	22.2(6)	13.3(6)	10.9(7)	0.0(5)	5.2(5)	-1.1(4)
O3	12.8(5)	9.1(5)	8.4(6)	-0.5(5)	1.6(5)	-0.8(4)
O4	19.5(5)	9.5(5)	10.7(7)	0.2(5)	6.0(5)	-1.3(4)
O5	19.9(6)	9.0(5)	13.9(7)	-1.2(5)	3.6(5)	-0.1(4)
O6	17.0(6)	12.3(5)	11.8(6)	3.5(5)	1.2(5)	1.2(4)
O7	15.5(5)	9.4(5)	13.4(7)	-0.4(5)	1.4(5)	-1.1(4)
O8	16.8(6)	12.2(5)	10.7(6)	1.7(5)	0.5(5)	-0.5(4)
O9	17.6(6)	19.7(6)	17.5(7)	0.5(6)	0.7(5)	-2.1(5)
C1	10.5(7)	12.1(7)	10.5(9)	1.9(6)	-0.7(6)	-2.1(5)
C2	9.4(7)	13.6(7)	7.0(8)	-0.4(7)	0.4(5)	-0.6(5)
C3	10.7(7)	13.5(7)	9.0(9)	-0.5(7)	1.4(6)	0.0(5)
C4	11.1(6)	10.6(6)	10.8(9)	-0.7(7)	-1.2(6)	0.1(5)
C5	11.2(7)	12.1(7)	8.7(8)	0.9(6)	-0.3(7)	-1.1(6)
C6	11.8(7)	13.0(7)	6.5(8)	0.1(7)	1.2(6)	-1.1(6)
Li1	18.2(14)	11.7(13)	13.9(17)	0.2(12)	-0.9(11)	-0.2(10)

Table S7. Bond Lengths for lithium comenate tetrahydrate $[Li(HCom)(H_2O)_3]\cdot H_2O$

Atom	Atom	Length/Å	Atom	Atom	Length/Å
O1	C1	1.254(2)	O6	Li1	1.994(3)
O2	C1	1.250(2)	O7	Li1	2.070(4)
O3	C2	1.346(2)	O8	Li1	2.030(3)
O3	C6	1.361(2)	C1	C2	1.512(2)
O4	C5	1.346(2)	C2	C3	1.354(2)
O4	Li1	2.109(3)	C3	C4	1.437(2)
O5	C4	1.2533(19)	C4	C5	1.445(2)
O5	Li1	2.157(3)	C5	C6	1.360(2)

Table S8. Bond angles for lithium comenate tetrahydrate $[Li(HCom)(H_2O)_3]\cdot H_2O$

Atom	Atom	Atom	Angle/°	Atom	Atom	Atom	Angle/°
C2	O3	C6	118.99(13)	O4	C5	C6	124.34(15)
C5	O4	Li1	112.37(14)	C6	C5	C4	120.71(16)
C4	O5	Li1	111.67(13)	C5	C6	O3	121.95(15)
O1	C1	C2	118.12(16)	O4	Li1	O5	76.55(11)
O2	C1	O1	127.29(16)	O6	Li1	O4	143.68(17)
O2	C1	C2	114.59(14)	O6	Li1	O5	87.88(13)
O3	C2	C1	113.33(13)	O6	Li1	O7	95.18(13)
O3	C2	C3	123.14(15)	O6	Li1	O8	115.35(16)
C3	C2	C1	123.50(16)	O7	Li1	O4	85.62(13)
C2	C3	C4	120.37(16)	O7	Li1	O5	153.22(17)
O5	C4	C3	125.14(16)	O8	Li1	O4	100.14(13)
O5	C4	C5	120.00(16)	O8	Li1	O5	102.71(15)
C3	C4	C5	114.83(14)	O8	Li1	O7	99.96(14)
O4	C5	C4	114.93(14)				

Table S9. Torsion Angles for lithium comenate tetrahydrate $[Li(HCom)(H_2O)_3]\cdot H_2O$

A	B	C	D	Angle/°	A	B	C	D	Angle/°
O1	C1	C2	O3	3.5(2)	C2	C3	C4	C5	1.2(2)
O1	C1	C2	C3	-174.92(15)	C3	C4	C5	O4	-178.69(14)
O2	C1	C2	O3	-177.16(13)	C3	C4	C5	C6	-0.2(2)
O2	C1	C2	C3	4.4(2)	C4	C5	C6	O3	-0.6(2)
O3	C2	C3	C4	-1.5(2)	C6	O3	C2	C1	-177.70(13)
O4	C5	C6	O3	177.77(14)	C6	O3	C2	C3	0.7(2)
O5	C4	C5	O4	-0.5(2)	Li1	O4	C5	C4	16.13(18)
O5	C4	C5	C6	177.97(15)	Li1	O4	C5	C6	-162.28(16)
C1	C2	C3	C4	176.74(14)	Li1	O5	C4	C3	163.02(15)
C2	O3	C6	C5	0.3(2)	Li1	O5	C4	C5	-15.0(2)
C2	C3	C4	O5	-176.87(15)					

Table S10. Hydrogen Atom Coordinates (Å×10^4) and Isotropic Displacement Parameters (Å^2×10^3) for lithium comenate tetrahydrate $[Li(HCom)(H_2O)_3]\cdot H_2O$

**Atom**	***x***	***y***	***z***	**U(eq)**
H6A	628.72	5468.45	2584.67	16
H6B	-261.72	5221.06	4164.61	16
H7A	3698.39	4943.2	276.77	15
H7B	3400.67	4333.58	-227.97	15
H8A	4668.11	4080.38	5347.39	16
H8B	5473.39	4158.98	3553.73	16
H4	2207.77	3301.87	1182.29	22
H9A	7745.01	4147.48	822.32	22
H9B	6132.28	3844.9	297.05	22
H3	-983.71	3211.06	7999.07	13
H6	1449.99	2286.54	2305.64	13

Table S11. Hydrogen Bonds for lithium comenate tetrahydrate $[Li(HCom)(H_2O)_3]\cdot H_2O$

**D**	**H**	**A**	**d(D-H)/Å**	**d(H-A)/Å**	**d(D-A)/Å**	**D-H-A/°**
O6	H6A	O5^1	0.85	1.91	2.7510(18)	169.3
O6	H6B	O7^2	0.85	1.98	2.8073(17)	163.6
O7	H7A	O8^3	0.85	1.97	2.7825(16)	160.6
O7	H7B	O1^4	0.85	2.04	2.8702(17)	165.0
O8	H8A	O1^5	0.85	1.97	2.8063(19)	167.6
O8	H8B	O9	0.85	1.95	2.789(2)	166.8
O4	H4	O2^4	0.89	1.64	2.5251(17)	170.8

Figure S1. TG-, DTG-, DSC-curves of a complex compound $[Li(HCom)(H_2O)_3]\cdot H_2O$

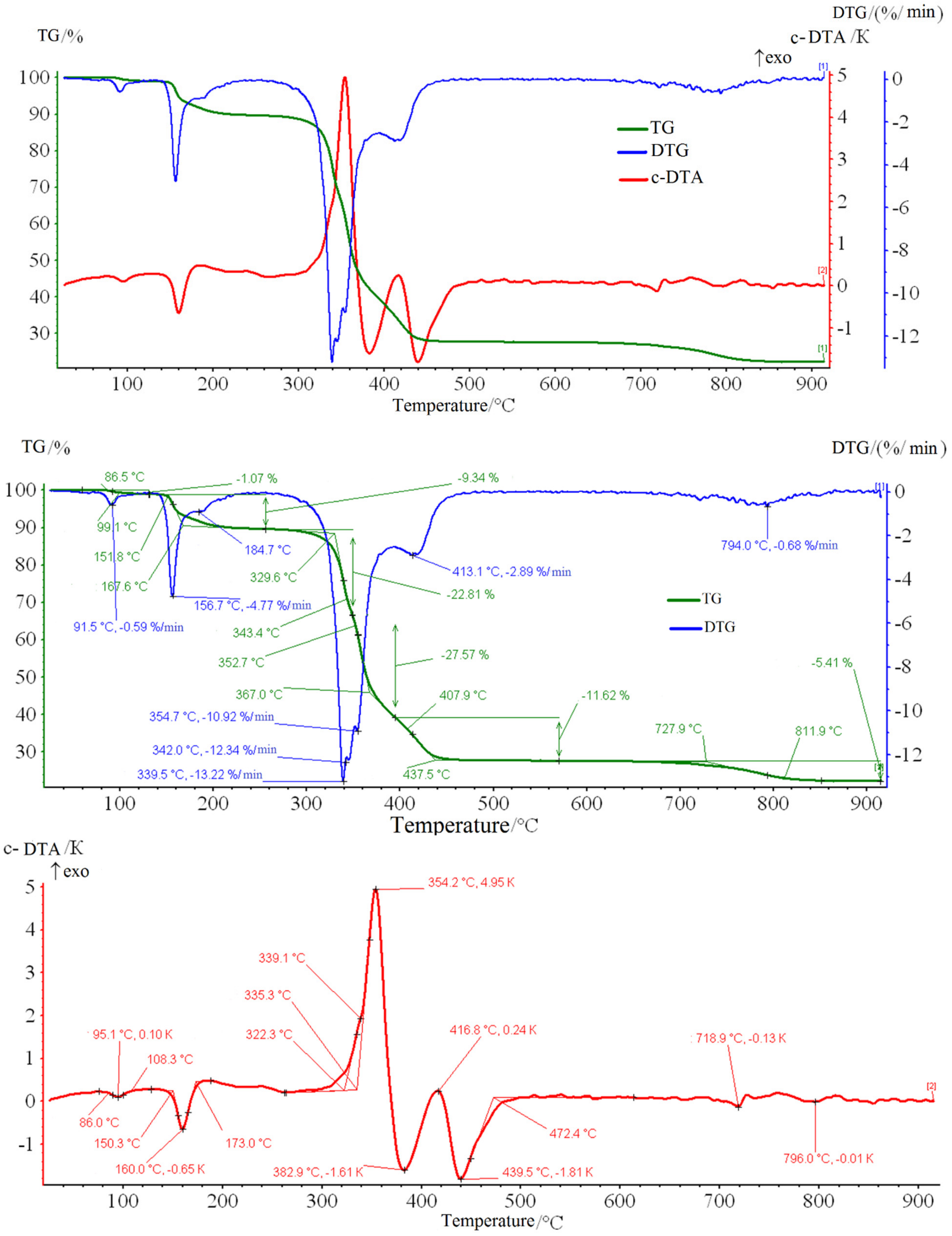

Figure S2. Non-valent intermolecular contacts in a lithium comenate crystal

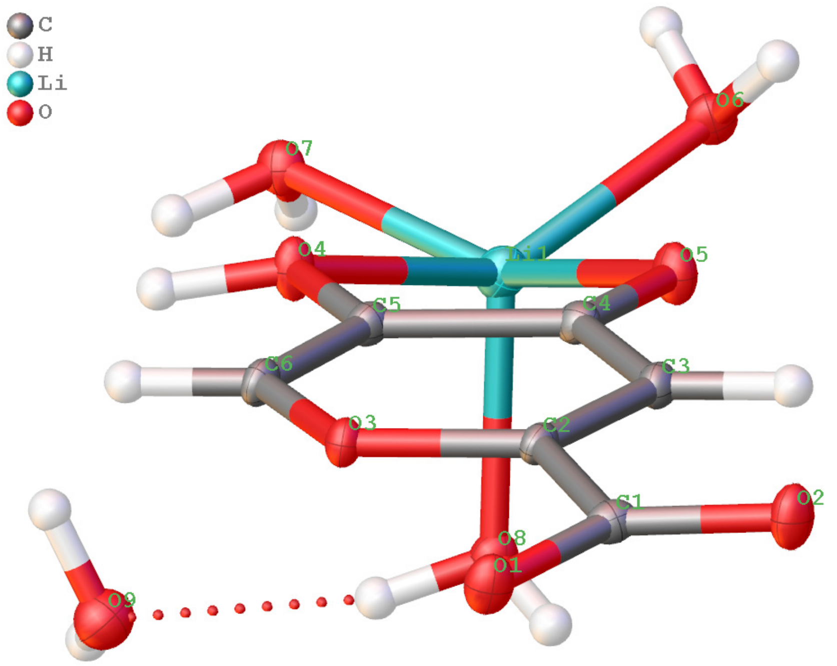

Figure S3. ^{1}H NMR (400 MHz, D_2O) spectrum of $[Li(HCom)(H_2O)_3]\cdot H_2O$

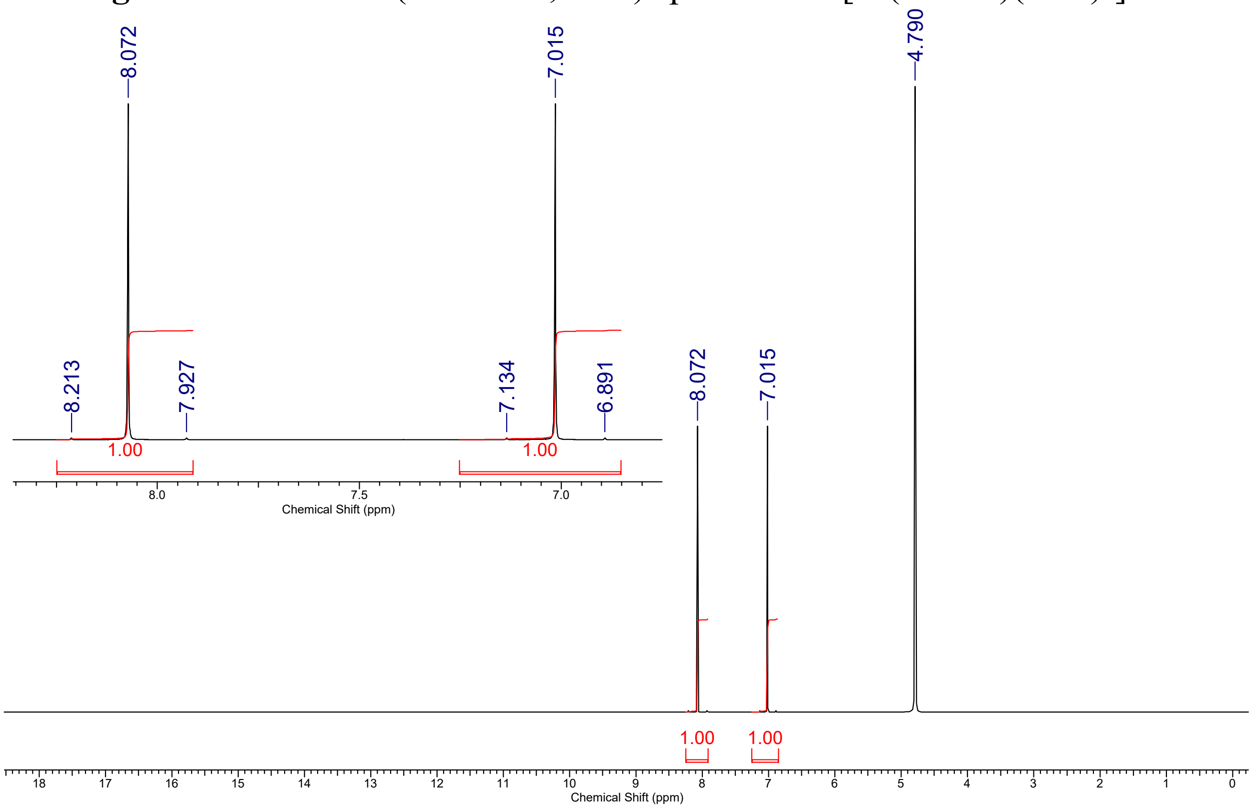

Figure S4. ^{13}C NMR (101 MHz, D_2O) spectrum of $[Li(HCom)(H_2O)_3]\cdot H_2O$

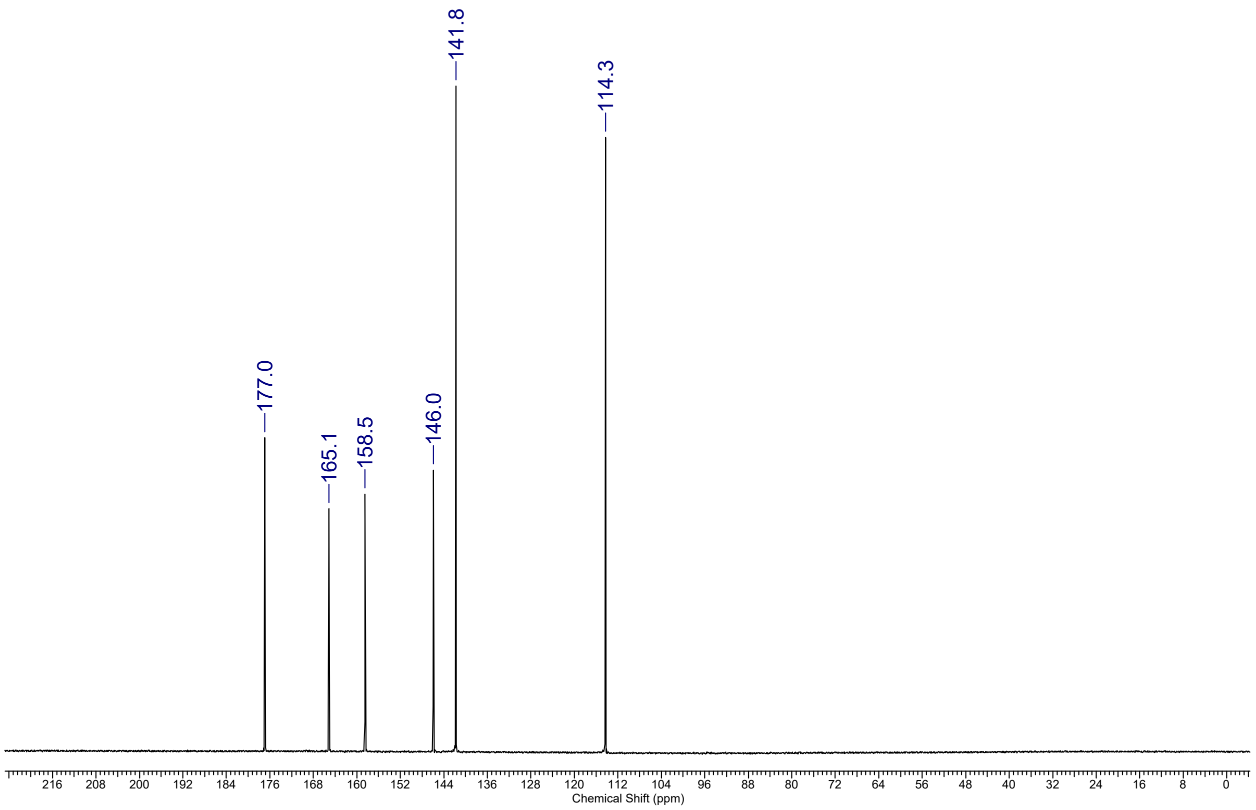

Figure S5. FTIR spectrum of comenic acid (ATR mode)

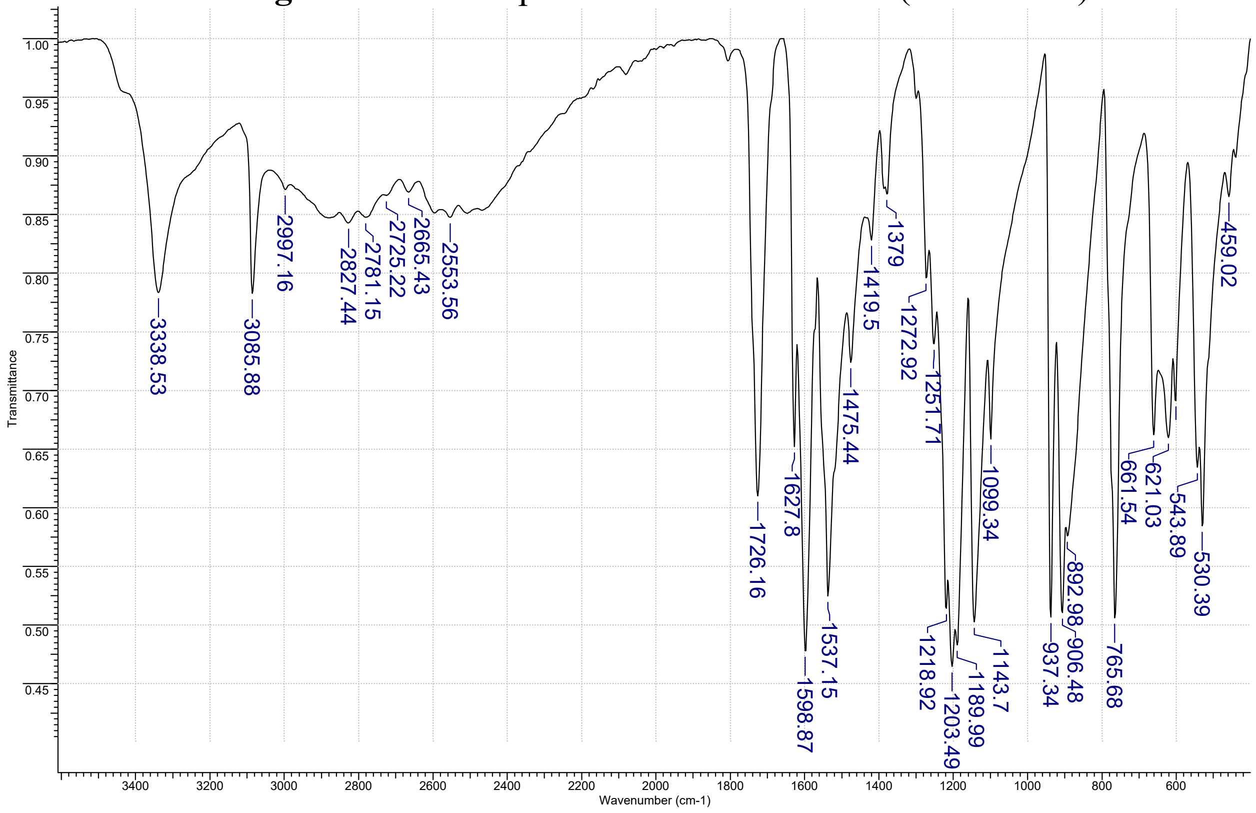

Figure S6. FTIR spectrum of complex compound $[Li(HCom)(H_2O)_3]\cdot H_2O$ (ATR mode)

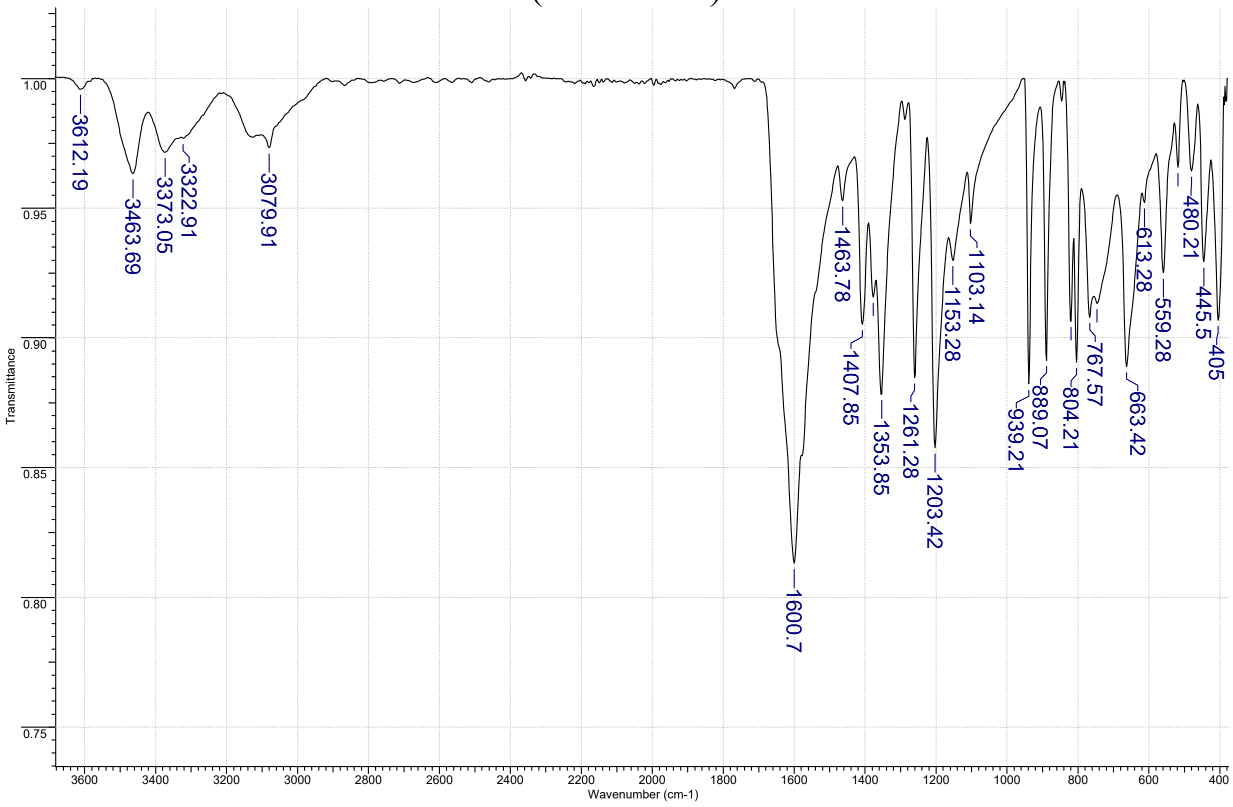

Supplement: Supplementary file 1 [file ijms-25-00286-s001.zip › ijms-2778045-supplementary.pdf]
